# A Simple Risk Model to Predict Survival in Patients With Carcinoma of Unknown Primary Origin

**DOI:** 10.1097/MD.0000000000002135

**Published:** 2015-10-30

**Authors:** Chen-Yang Huang, Chang-Hsien Lu, Chan-Keng Yang, Hung-Chih Hsu, Yung-Chia Kuo, Wen-Kuan Huang, Jen-Shi Chen, Yung-Chang Lin, Hung Chia-Yen, Wen-Chi Shen, Pei-Hung Chang, Kun-Yun Yeh, Yu-Shin Hung, Wen-Chi Chou

**Affiliations:** From the Division of Hematology and Oncology, Department of Internal Medicine, Chang Gung Memorial Hospital at Linkou and Chang Gung University School of Medicine, Taoyuan (C-YH, C-KY, H-CH, Y-CK, W-KH, J-SC, Y-CL, C-YH, W-CS, Y-SH, W-CC); Division of Hematology and Oncology, Department of Internal Medicine, Chang Gung Memorial Hospital, Chiayi (C-HL); Division of Hematology and Oncology, Department of Internal Medicine, Chang Gung Memorial Hospital, Keelung (P-HC, K-YY); and Graduate Institute of Clinical Medical Sciences, College of Medicine, Chang Gung University, Taoyuan, Taiwan (W-CC).

## Abstract

Carcinoma of unknown primary origin (CUP) is characterized by diverse histological subtypes and clinical presentations, ranging from clinically indolent to frankly aggressive behaviors. This study aimed to identify prognostic factors of CUP and to develop a simple risk model to predict survival in a cohort of Asian patients.

We retrospectively reviewed 190 patients diagnosed with CUP between 2007 and 2012 at a single medical center in Taiwan. The clinicopathological parameters and outcomes of our cohort were analyzed. A risk model was developed using multivariate logistic regression and a prognostic score was generated.

The prognostic score was calculated based on 3 independent prognostic variables: the Eastern Cooperative Oncology Group (ECOG) scale (0 points if the score was 1, 2 points if it was 2–4), visceral organ involvement (0 points if no involvement, 1 point if involved), and the neutrophil-to-lymphocyte ratio (0 points if ≤3, 1 point if >3). Patients were stratified into good (score 0), intermediate (score 1–2), and poor (score 3–4) prognostic groups based on the risk model. The median survival (95% confidence interval) was 1086 days (500–1617, n = 42), 305 days (237–372, n = 75), and 64 days (44–84, n = 73) for the good, intermediate, and poor prognostic groups, respectively. The c-statistics using the risk model and ECOG scale for the outcome of 1-year mortality were 0.80 and 0.70 (*P* = 0.038), respectively.

In this study, we developed a simple risk model that accurately predicted survival in patients with CUP. This scoring system may be used to help patients and clinicians determine appropriate treatments.

## INTRODUCTION

Carcinoma of unknown primary origin (CUP) is defined as histologically proven metastatic carcinoma whose primary site cannot be identified despite a complete history review, physical examination, and diagnostic evaluation.^1^ CUP is an uncommon disease in clinical practice, representing 3% to 5% of all invasive malignancies.^1^ A wide variation in the prevalence rate of CUP has been reported worldwide, with a spectrum ranging from 4 to 6 cases per 100,000 people per year in Switzerland to 18 to 19 cases per 100,000 people per year in Australia.^2^ Since a uniform definition and consensus is lacking in the CUP registry, the real incidence of CUP is unknown. Nevertheless, the prevalence rate of CUP has declined gradually in some cohort series;^3–7^ this decrease might be attributed to improvements in imaging tools and pathologic diagnoses.^4,5^

CUPs occur in heterogeneous patient groups with different clinical presentations, natural courses, and histological tumor types. The majority of patients often present with disseminated tumors involving multiple organs, while some patients present with a solitary tumor limited to a single anatomic location. As it is a metastatic disease entity, the outcome of patients with CUP is usually pessimistic, with a median survival of around 3 to 8 months.^8–14^ However, a small subset of patients with CUP has extended survival, probably because of an indolent clinical course, a combination of multidisciplinary treatment modalities, or an excellent response to antitumor treatment.^15^

Due to the heterogeneous clinical presentation and outcome of CUPs, appropriate patient selection and management might help to identify subsets of patients with more favorable outcomes. Several previous studies have reported clinical variables, including histological type,^16–18^ metastatic site,^10,12,13,16,19–21^ and functional performance of the patients,^9,11,12,20–24^ having significant impact on survival in patients with CUPs. However, all of the previous studies were limited to Western populations; data regarding the prognostic factors in Asian populations with CUPs are very limited. The present study aimed to identify the prognostic factors and to develop a prognostic score for predicting the outcome of patients with CUPs.

## METHODS

### Patient Selection

Two hundred eighty-six consecutive patients with a diagnosis of CUP in Chang Gung Memorial Hospital at Linkou between January 2007 and December 2012 were collected. All patients received at least a cytopathological diagnosis of carcinoma and were categorized as CUP based on the judgment of their primary care physician, who failed to identify the patient's primary cancer after a detailed physical examination and imaging study including chest and abdomen computed tomography (CT) scans, as well as endoscopic examination in selected patients. After a retrospective chart review for all patients, 96 patients were excluded from the study because of a lack of pathologic confirmation (eg, suspicion of malignancy or dysplasia, n = 48), noncarcinoma histological type (n = 26), inadequate CT scan workup (n = 12), and primary tumor identified after diagnostic examination (n = 10). In total, 190 patients were included in the final analysis. The study was approved by the ethics committee of the institute.

### Data Collection

Data on patient demographics, body mass index, tobacco use, tumor histological type, tumor grade, organ with tumor involvement, Eastern Cooperative Oncology Group performance (ECOG) score, peripheral complete blood counts at the time of CUP diagnosis, the use of antitumor therapy after CUP diagnosis, and the survival time were retrospectively obtained from medical charts. The treatment strategy included all antitumor therapies to a patient after the diagnosis of CUP. And the number of metastatic sties was defined as the number of organs found with tumor metastases at the time of CUP diagnosis. Therefore, the sum (combined) percentage in each category may exceed 100%. Visceral organ involvement is defined as a tumor metastasizing to any one of the following sites: liver, lung, peritoneum, bone marrow, or central nervous system. The neutrophil-to-lymphocyte ratio (NLR) was calculated by dividing the blood neutrophil count by the blood lymphocyte count. The survival time was calculated from the date of pathologic diagnosis of CUP to the date of death. All patients were followed up until death or December 31, 2014. The date of death was obtained through either the institutional Cancer Center registry system or the National Registry of Death database in Taiwan.

### Statistical Analysis

Basic demographic data were summarized as the n (%) for categorical variables and medians with ranges or 95% confidence interval (CI) for continuous variables. Twelve predefined variables recorded at the time of the diagnosis of CUP were then evaluated to ascertain their impact on patient survival. These key potential prognostic variables were selected because minimal data were missing and they could represent widely available clinical data, making the findings broadly applicable. An a priori statistical analysis plan was approved in univariate analysis; 7 of 12 variables with a *P* value <0.10 in univariate analysis were included for analysis in the multivariate model. A multivariate, proportional hazard Cox model with backward selection was performed to determine which factors were independently predictive of survival. A risk model was developed from a multivariate logistic regression. The *β* coefficients from the risk model were used to generate the points of the prognostic score for calculating survival time. Receiver operating characteristic (ROC) curves and the area under the curve (c-statistic) for the outcome of overall survival at 1, 2, and 3 years were calculated to determine the accuracy of the prognostic score.

Patients were further stratified into 3 prognostic groups according to the total score obtained from the prognostic score. Overall survivals among different prognostic categories were calculated according to the Kaplan–Meier method. Log-rank tests were used to determine significant differences among the survival curves. SPSS 17.0 software (SPSS Inc, Chicago, IL) was used for statistical analysis. All statistical assessments were 2-sided. A *P* value less than 0.05 was considered significant.

## RESULTS

Table 1 shows the demographic data of the 190 patients. The median age was 61 years (range, 12–91) and 59.5% of the patients were male. One hundred twelve patients (58.9%) had an excellent ECOG score (0 or 1). Undifferentiated carcinoma was the most common histological subtype (64 patients, 33.7%), followed by adenocarcinoma (60 patients, 31.6%), squamous cell carcinoma (43 patients, 22.6%), and neuroendocrine carcinoma (19 patients, 10%). The distribution of the number of metastatic organ sites with 1, 2, and 3 or more was 51.1%, 25.8%, and 23.2% of all patients, respectively. The most common metastatic site was the lymph nodes (66.3%), followed by the liver (26.3%), lung (24.7%), bone (22.6%), peritoneum (20.0%), central nervous system (4.7%), bone marrow (2.6%), and the other sites (15.8%). Regarding the treatment strategy of all 190 patients with CUPs, 113 patients (59.5%) received systemic chemotherapy, 86 patients (45.3%) received radiotherapy, 45 patients (23.7%) received surgical debulking, and 6 patients (3%) received other miscellaneous treatments. Furthermore, 37 patients (19.5%) received the best supportive care without antitumor treatment.

**TABLE 1:**
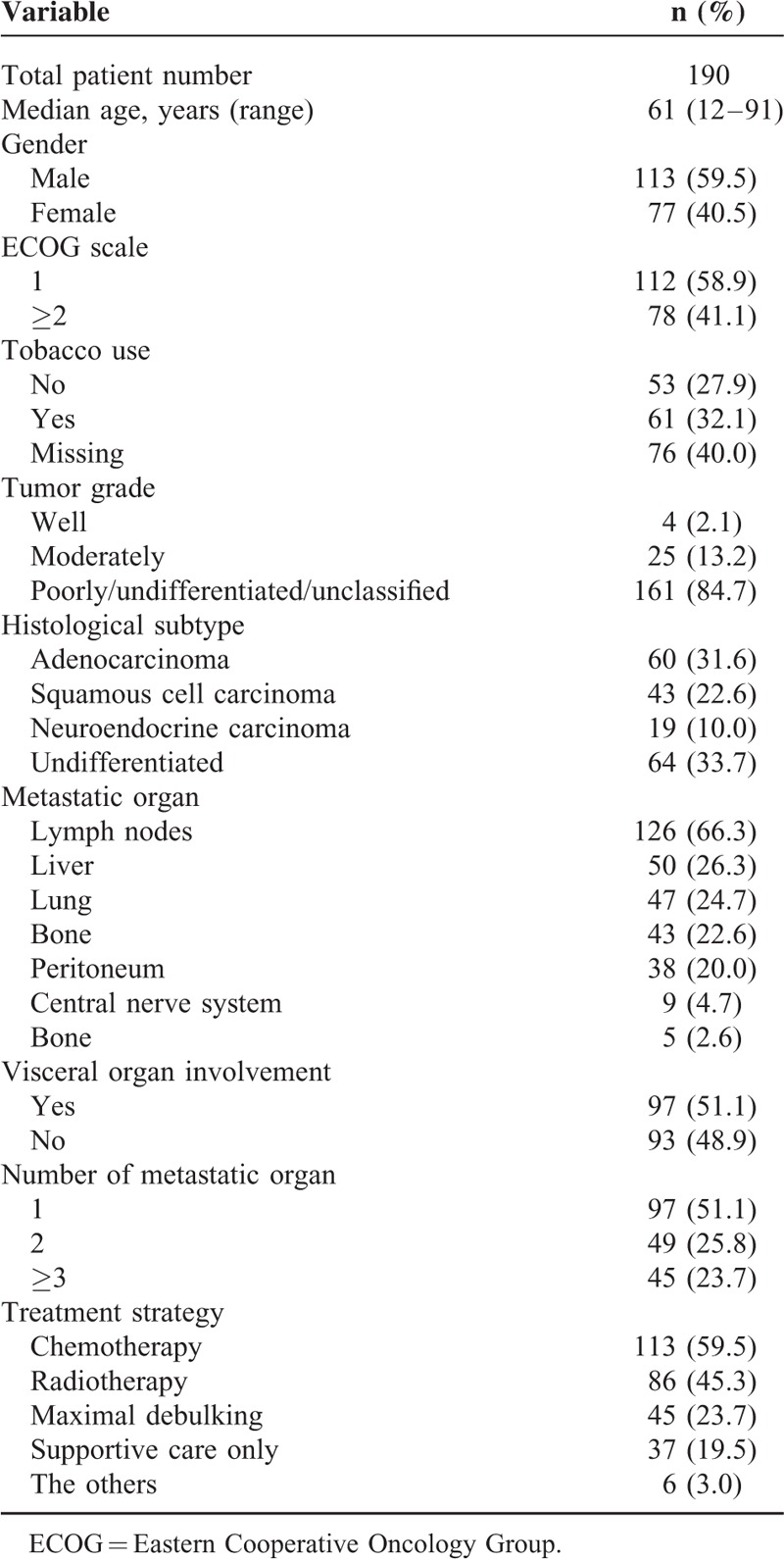
Basic Demographic Data of Patients

In order to test whether a specific treatment affected a patient's prognosis, we analyzed the median survival time by stratified patients into different groups based on the main treatment one received (Table 2). The median survival time was the shortest of only 1.2 month in patients who received supportive care only and highest of 68.4 among those who had maximal debulking surgeries along with chemoradiotherapy. In an ascending order, the median survival for the remaining groups is 4.8, 6.8, 10.9, 35.7, and 60.6 months in radiotherapy only, chemotherapy only, chemoradiotherapy, maximal debulking surgery only, and maximal debulking surgery with either chemotherapy or radiotherapy, respectively. Patients received any treatment other than support care only had a significantly longer median survival time (*P* < 0.001).

**TABLE 2:**
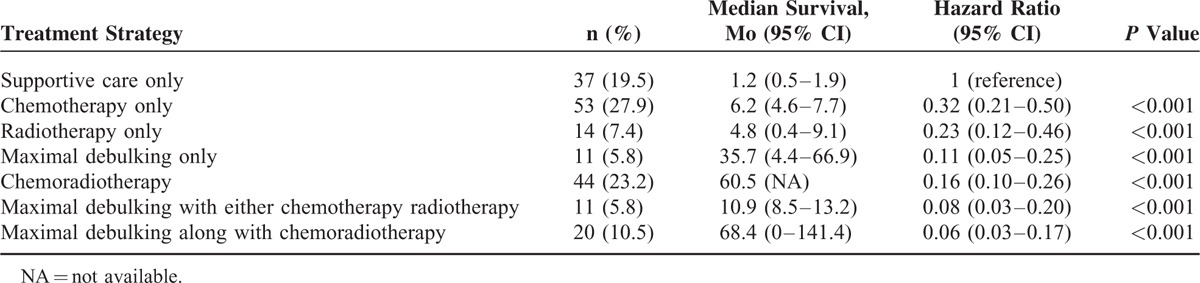
Survival Times of Patients Receiving Different Main Treatment Strategies

The median survival time was 6.8 months (range, 0.1–78.9). At the end of the follow-up period, 157 patients (82.6%) had died. The 1-, 2-, and 3-year survival rates were 38%, 26%, and 19%, respectively. The results of univariate and multivariate analyses of survival that were associated with the clinical variables are presented in Table 3. Based on univariate analysis of the imputed data set, 7 of the 12 preselected variables, including age, number of metastatic organs, visceral organ involvement, ECOG score, hemoglobin level, platelet count, and NLR, showed a statistically significant effect on survival. However, multivariate analysis identified ECOG scale, visceral organ involvement, and NLR as the only independent prognostic factors.

**TABLE 3:**
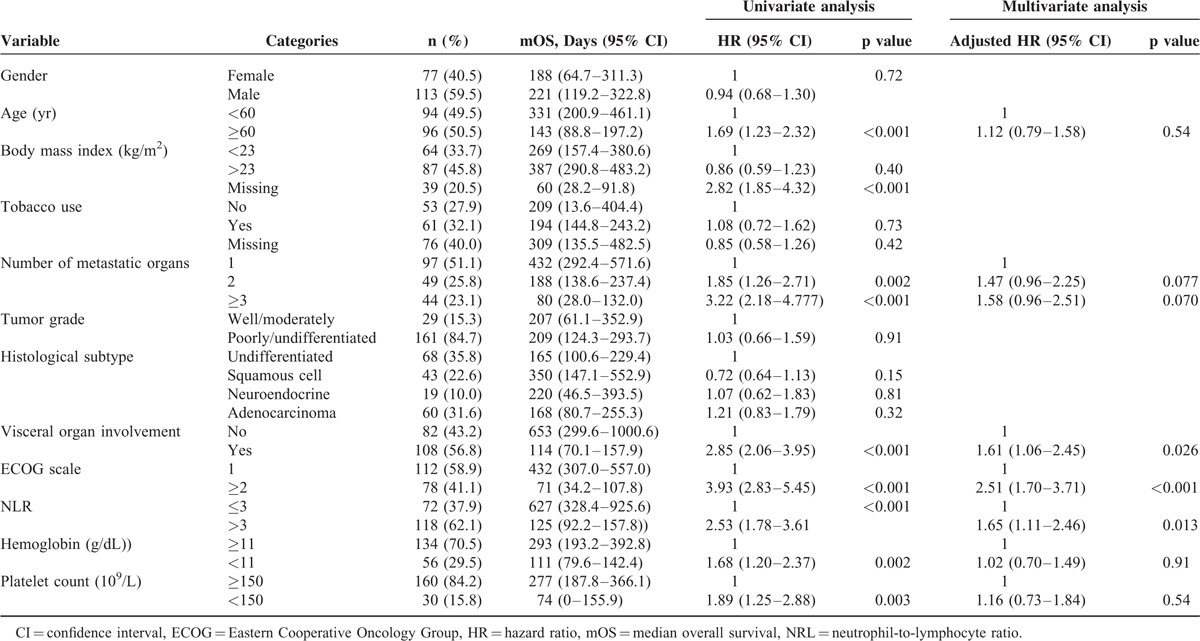
Univariate Analysis and Multivariate Analysis for Overall Survival

**FIGURE 1:**
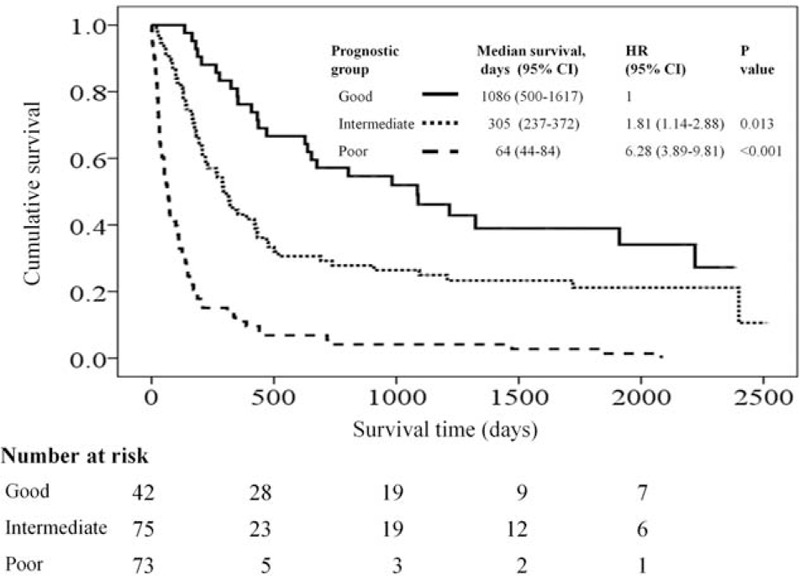
Kaplan–Meier survival curves for patients stratified by prognostic groups.

The risk model and scoring system of prognostic score generated from *β* coefficients of multivariate analysis are shown in Table 4. The total prognostic scores ranged from 0 to 4. Using the prognostic score, patients were stratified into good (sum score 0), intermediate (sum score 1–2), and poor (sum score 3–4) prognostic groups. The prognostic score assigned 22.1% of the patients to the good, 39.5% to the intermediate, and 38.4% to the poor prognostic groups. Accordingly, the median survival times of different prognostic groups are shown in Figure 1. The median (95% CI) survival in the good, intermediate, and poor prognostic risk groups was 1086 days (500–1617), 305 days (237–372), and 64 days (44–84), respectively. The hazard ratios were 1.18 (95% CI, 1.14–2.88; *P* = 0.013) when comparing the intermediate and good prognostic groups and 6.28 (95% CI, 3.89–9.81; *P* < 0.001) when comparing the poor and good prognostic groups.

**TABLE 4:**
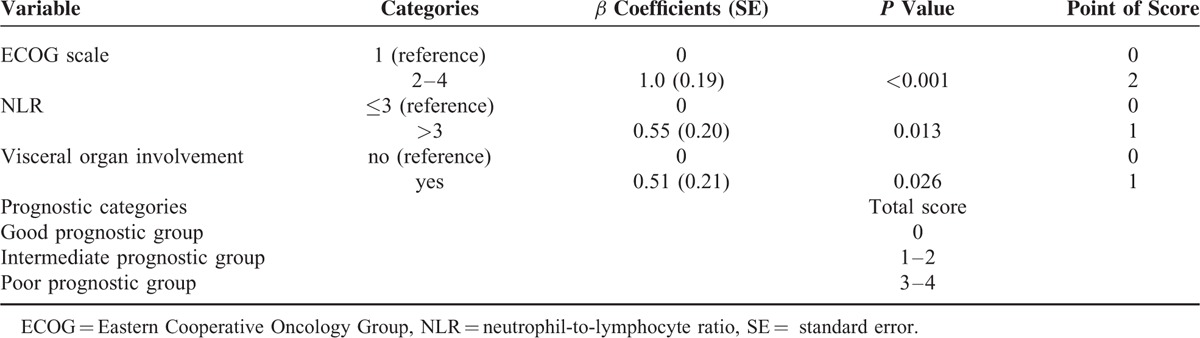
Risk Model and Prognostic Score

**FIGURE 2:**
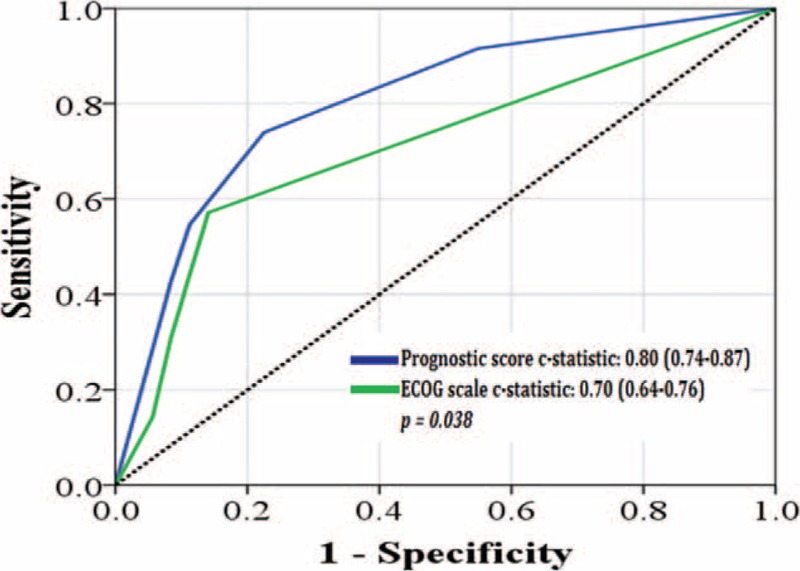
Receiver operating characteristic analysis using the prognostic score (blue line) and Eastern Cooperative Oncology Group (ECOG) scale (green line) for the outcome of 1-yr mortality.

The ROC curve analysis for mortality at 1, 2, and 3 years using the prognostic score gave significantly higher c-statistic values than the ECOG score alone. The c-statistic at 1 year was 0.80 (95% CI, 0.74–0.87) for the prognostic score compared with 0.70 (95% CI, 0.64–0.76) for the ECOG score (*P* = 0.038) (Figure 2). At 2 years, the c-statistic for the prognostic score was 0.80 (95% CI, 0.73–0.87) compared with 0.69 (95% CI, 0.61–0.78) for the ECOG score (*P* = 0.009). At 3 years, the c-statistic for prognostic score was 0.77 (95% CI, 0.69–0.84) compared with 0.68 (95% CI, 0.61–0.78) for the ECOG score (*P* = 0.041).

## DISCUSSION

This study identified a new prognostic score and generated a risk model that predicts survival in patients with CUP at a medical center over 6 years in Taiwan. The diagnosis of CUP is usually indicative of disseminated cancer, a rapidly deteriorating clinical course and a dismal prognosis. In general, the outcome of patients with CUP is usually dismal, with a median survival time ranging from 3.4 to 16.5 months (Table 5). Some reasons may partially explain the variability in survival times among different reports. First, there is no consensus about the definition of CUP; tumors with the same histological type in the same organ may be categorized differently according to the experience of the primary care physician or the interpretation of the pathologic report. Second, with the improvement of imaging tools and pathologic diagnoses, tumors originally categorized as CUP may be more likely to receive treatment as most-possible chemosensitive cancer types. Third, the treatment strategies and medical care may change between different study periods and medical resources. The clinical outcome of this study closely resembled previous reports from Western countries.^8–14,21^ Nevertheless, this study found that a small subset of patients with CUPs (19%) survived longer than 3 years. Accurate prognostic stratification of CUP may assist clinicians in counseling patients appropriately and selecting patients who are likely to benefit from antitumor treatments.

**TABLE 5:**
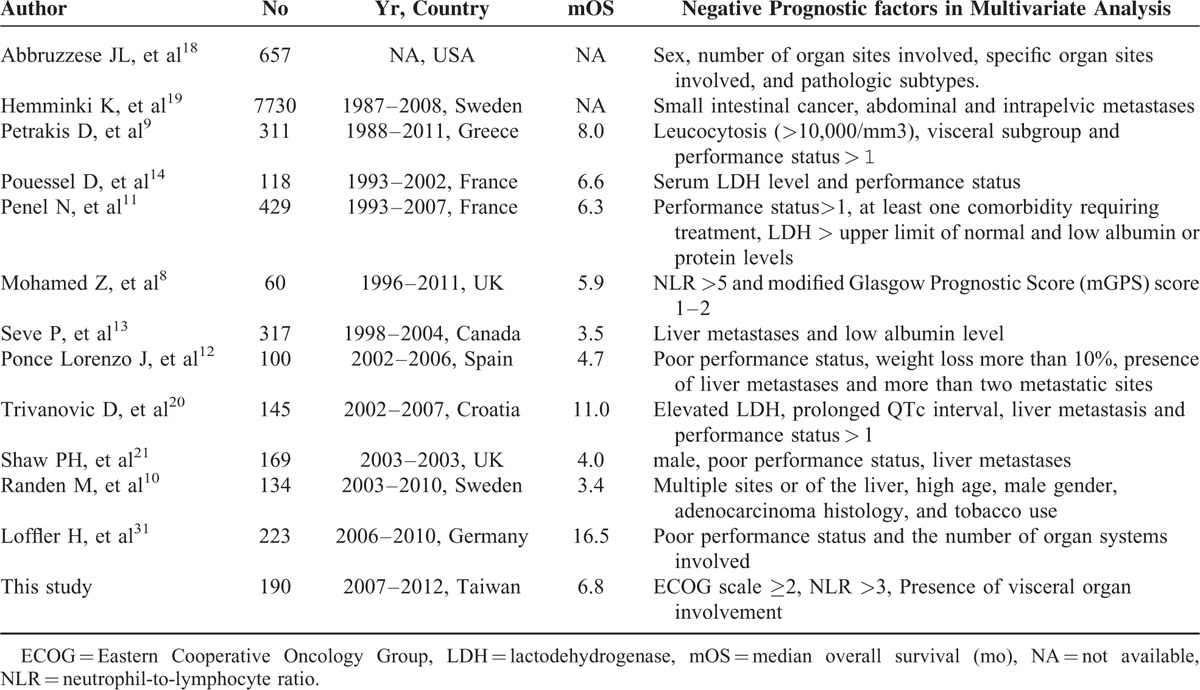
Comparison of Different Retrospective Review of Patients With CUP and Prognosis

Previous studies have aimed to identify prognostic factors in patients with CUP. Gender,^21^ performance status,^9,11,12,20–24^ and number of organs involved and histological subtype ^16–18^ were the most frequently reported prognostic factors for patients with CUP in Western countries. This study identified that the independent predictors of survival time were visceral organ involvement, ECOG score and NLR in an Asian population. The clinical variables of this risk model are accessible and are available at the time of diagnosis. Therefore, this model is widely applicable and clinically relevant.

The involvement of visceral organs indicated an aggressive tumor course, a distantly disseminated tumor and a poor functional reserve of vital organs. The ECOG scale was originally designed to determine whether a patient was eligible to receive chemotherapy,^25^ and it is now widely used to determine eligibility for clinical trials, measure quality of life, predict treatment-related toxicities, and estimate prognosis in oncology practices across different cancer types.^26–31^ The ECOG score contributed as a prognostic factor in patients with CUP since it represents the general health of the patient and quantifies the activities of daily life.^23,31^ In Table 5, we summarized known poor prognostic factors from selected retrospective studies. These factors can be divided into 2 major categories. First category is a patient's demographics, including gender, age, tobacco usage, performance status, and blood cell counts. Second is a patient's disease status, that is the number of metastatic sites, visceral organs involvement, and tumor burden. Our study showed that this risk model combining the patient's demographic with ECOG score and neutrophil-to-lymphocyte ratio as well as the disease status with visceral organ involvement could provide better risk stratification of survival times than the ECOG score alone.

The NLR is an emerging marker of host inflammation and has been shown to be an independent prognostic factor in numerous epidemiologic studies of various cancer types.^32^ Higher NLR has been found to be consistently associated with more advanced stage and more aggressive tumor behavior.^33^ A putative mechanism hypothesizes that lymphocytopenia would negatively regulate tumor defenses by inducing cytotoxic cell death and inhibiting tumor proliferation.^34,35^ In contrast, increased neutrophil counts may decrease the cytolytic activity of lymphocytes or natural killer cells and could promote tumor growth.^36,37^ More recently, Mohamed et al reported that inflammatory markers with NLR and Glasgow Prognostic scores were both independent prognostic factors and were superior to ECOG score in patients with CUPs.^8^ Similarly, this study confirmed the prognostic value of NLR in patients with CUPs. Compared with other systemic inflammatory markers, such as C-reactive protein ^38^ and the Glasgow Prognostic Score,^39^ NLR is easily calculated from routine complete blood counts with differentials, which is an essential blood test in cancer patients.

The use of antitumor therapy is a positive prognostic factor in patients with CUP.^40^ However, we did not include this variable in the survival analysis because it could be confounded by other variables, such as performance status, histological subtype, metastatic site, and the clinicians’ preferences. Therefore, this variable could not be used immediately after CUP was diagnosed. From a practice point of view, estimating an individual patient's outcome immediately after a diagnosis of CUP could provide valuable prognostic information to the clinician, patients, and their families. To preserve the utility of this model for decision making, we used only the clinical variables that are easily accessed and available at the time of CUP diagnosis. Neither the cancer type, specific laboratory value, type of treatment strategy, nor the post-treatment variables were added for analysis, despite the fact that these could influence mortality. Therefore, this model could be used in routine clinical practice to predict outcomes in all patients with a new diagnosis of CUP.

The optimal systemic treatment for CUP has not been established. Platinum-based chemotherapy in combination with paclitaxel, docetaxel, gemcitabine, and irinotecan has been reported to have objective response rates ranging from 18% to 55%.^41–45^ Although there is no standard treatment for CUP, proper management can still lead to long-term survivorship. Kaizu et al demonstrated that definitive chemoradiation with platinum-based chemotherapy for cervical lymph node metastases from CUP was well tolerated, with a 5-year overall survival of 52%.^46^ We believe that this prognostic score is important to aid in the decision-making process for patients and clinicians regarding the treatment strategy of CUP. Patients with a good prognosis may be encouraged to undergo more aggressive and multidisciplinary antitumor therapies. In contrast, for patients with a particularly poor prognosis, given their median survival of only 2 months in our study, antitumor therapy may represent a futile treatment. Appropriate end-of-life care should be provided for patients categorized into the poor prognostic group.

The strengths of our study included large patient numbers from a single institute in Taiwan over a 6-year duration. To the best of our knowledge, our study is the first to develop a prognostic model in Asian populations of patients with CUP. However, this study had several limitations. First, a selection bias may exist, as this was a retrospective study. Second, the clinical practice and healthcare system in Taiwan may differ from other countries. Therefore, this model may not be applied to all cancer patients worldwide. It is essential that this risk model be validated externally in other countries before it can be used in clinics worldwide. In a population-based study, Urban et al reported a higher incidence of CUP among elderly, females, blacks, and those from less affluent or educated counties,^6^ while Shu et al found a lower incidence of CUP among Asian immigrants in Sweden.^7^ In our study, all patients were Taiwanese with a relatively similar life style. It would be difficult to draw any conclusion from our result to address the potential impact of race and environment on CUP incidence in our study. A multicountry, multivariate analysis on all published data would be helpful to identify if certain ethnic group or environment is more likely put a patient at risk of CUP. Third, our study included a heterogeneous patient group with respect to antitumor treatment modalities; as such, there was selection bias regarding which patients were offered the treatment, and the effectiveness of the antitumor therapy may also potentially affect patient outcome.

In conclusion, this study developed a simple risk model that accurately predicted survival in patients with CUP. This scoring system may be used to help patients and clinicians in deciding appropriate treatments.
